# Diagnostic utility of Xpert MTB/RIF ultra in clinically suspected tuberculous lymphadenitis in low-burden settings: a single center experience from Saudi Arabia

**DOI:** 10.1016/j.jctube.2026.100603

**Published:** 2026-04-11

**Authors:** Manar Almutairi, Reema Alalmaee, Elaf Alamer, Shaden Alharbi, Gadah Aljarallah, Raghad Almukhayzim, Nawaf Albuhayjan, Reem Abanamy

**Affiliations:** a Department of Medicine, King Abdulaziz Medical City, Riyadh, Saudi Arabia; b King Abdullah International Medical Research Center (KAIMRC), Riyadh, Saudi Arabia

**Keywords:** Tuberculous lymphadenitis, Extrapulmonary tuberculosis, GeneXpert Ultra, Molecular diagnostics, Saudi Arabia

## Abstract

**Background:**

Tuberculosis (TB) is a major global health issue, particularly among HIV-positive individuals. In Saudi Arabia, the incidence has declined to 8.4 per 100,000 but remains a concern among high-risk groups. Extrapulmonary TB, especially tuberculous lymphadenitis (TBL), is challenging to diagnose due to the low sensitivity of smear, culture, and histopathology. Xpert MTB/RIF Ultra (Xpert-Ultra) offers improved detection in paucibacillary disease, though local evidence remains limited.

**Method:**

We retrospectively reviewed 85 adult patients with suspected TBL at King Abdulaziz Medical City, Riyadh (2012–2024). Clinical, radiological, histopathological, and microbiological data were collected. The sensitivity of Xpert-Ultra, GeneXpert, and ProbTec was assessed against a composite reference standard comprising confirmed and presumed TB cases.

**Result:**

Among 85 patients, the majority were males (55.3%) and Saudi nationals (91.8%), with most aged 41–65 years (36.5%). Cervical lymphadenopathy was the most common presentation (64.7%). Necrotizing granulomas were observed in 78.8% of biopsies. Culture was positive in 43.6% (17/39), while AFB staining was detected in 14.8% (12/81). Overall, PCR sensitivity was 38.8% (33/85). Xpert-Ultra achieved higher sensitivity (41.3%) compared with GeneXpert (31.6%) and ProbTec (33.3%). Xpert-Ultra missed two culture-positive cases but detected three culture-negative ones. PCR positivity was associated with extensive lymph node involvement and culture positivity.

**Conclusion:**

Xpert-Ultra outperformed older molecular assays but remained insufficient as a standalone diagnostic tool for TBL. Its complementary role alongside histopathology and culture supports its integration into diagnostic pathways, potentially improving time to initiation of treatment.

## 1 Introduction

Tuberculosis (TB) is an infectious disease caused by mycobacteria from the *Mycobacterium tuberculosis* complex. [1] It remains a major global health challenge, with an estimated 10.8 million people affected worldwide in 2023 and a global incidence rate of approximately 134 per 100,000 population. [1] Vulnerable groups are most affected, with individuals co-infected with HIV accounting for 6.1% of all TB cases. [1] In Saudi Arabia, TB incidence has declined to 8.4 per 100,000 in 2023, among the lowest in the region. However, it continues to pose a public health concern, particularly among migrant workers and immunocompromised individuals. [2] The highest TB prevalence was found in Riyadh, Dammam, and Jeddah, while the lowest prevalence was found in Jazan and Hail. [3] The incidence has been steadily declining over the last two decades; several Saudi studies and the national TB program report progress toward WHO End-TB milestones. [2].

Lymphadenitis is the most common presentation of extrapulmonary tuberculosis (EPTB). [4] The clinical features and outcomes of tuberculosis lymphadenitis (TBL) have been studied globally. The most common symptom is swelling of the lymph nodes, though other symptoms can vary. [4] Systemic symptoms like fever, weight loss, and night sweats are reported with different frequencies. [5] In Saudi Arabia, national data indicate that lymph node involvement accounts for approximately 42–56% of EPTB cases. [6], [7] Despite its high prevalence, microbiologic confirmation is often limited; in one Riyadh tertiary center, only 67.3% of TBL cases were culture-confirmed, indicating that a substantial number of cases are presumed based on histopathology or clinical criteria. [8] Regional histopathology-based studies report TBL in 8.8–14.2% of lymph node specimens. [7], [9].

Given the high prevalence of TBL, accurate diagnosis is critical. Conventional methods such as microscopy, culture, and histopathology have notable limitations due to the paucibacillary nature of TBL. [10] Microscopy, particularly acid-fast bacilli (AFB) staining, has low sensitivity, while culture, though considered the gold standard and useful for drug susceptibility testing, often takes 2–8 weeks. [10] Histopathological examination can reveal caseating granulomas, a hallmark of TB, but findings are not specific. [11] To overcome these limitations, the World Health Organization (WHO) recommended the use of the Xpert MTB/RIF Ultra (Xpert-Ultra) assay in 2017 for diagnosing TB in high-risk populations, including individuals with HIV, children, and those with extrapulmonary disease. [12] Xpert-Ultra is now preferred because it targets multicopy insertion sequences (IS6110, IS1081) in addition to the rpoB gene sequence, thereby lowering the detection threshold and increasing sensitivity compared to previous versions. [12].

Multiple studies have demonstrated that Xpert-Ultra exhibits higher sensitivity than Xpert MTB/RIF, smear microscopy, and culture techniques. [13], [14] Xpert-Ultra's sensitivity ranged from 67% to 85.4% across different sample types, including fine-needle aspirates (FNA) and tissue biopsies. [13], [14] A study conducted in India reported a sensitivity of 96.18% for Xpert Ultra in various extrapulmonary specimens, including lymph node biopsies, compared to 69.23% for the standard Xpert test. [15] Similarly, research in Tunisia found that Xpert Ultra achieved a sensitivity of 87.5% in lymph node aspirates and biopsies, surpassing the performance of traditional methods. [16] Notably, Xpert-Ultra increased the percentage of confirmed TB lymphadenitis cases from 55.4% to 85.1% when integrated into the diagnostic process. [13] These findings suggest that Xpert-Ultra is a valuable tool for the rapid and accurate diagnosis of TBL, particularly in high-burden settings.

Given these diagnostic challenges and the potential advantages of the Xpert MTB/RIF Ultra assay, this study aims to evaluate its diagnostic accuracy for detecting tuberculous lymphadenitis compared to clinical diagnosis in a low TB-burden, low HIV-prevalence setting at a tertiary care center in Saudi Arabia.

## 2 Methods

### 2.1 Study design and setting

A single-center retrospective cohort chart review study of adult patients (≥18 years) with suspected tuberculosis lymphadenitis, conducted at King Abdulaziz Medical City, an academic government-funded tertiary hospital in Riyadh, Saudi Arabia.

### 2.2 Study population

**Fig. 1:**
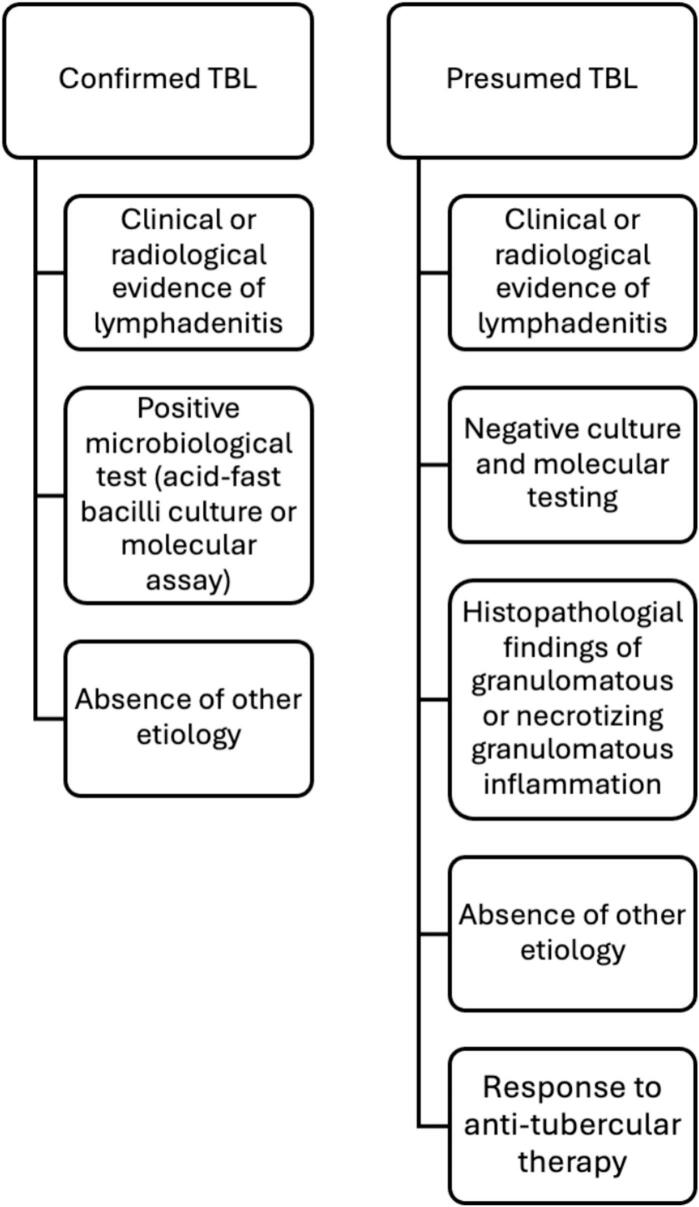
Composite reference standard used for classification of patients TBL: Tuberculous lymphadenitis.

Eligible participants included those 18 years of age and above, both inpatients and outpatients with clinical and pathological suspicion of TB lymphadenitis, who had undergone GeneXpert MTB/RIF Ultra or other molecular testing on their lymph node biopsy samples between 2012 and 2024. The sensitivity of GeneXpert MTB/RIF Ultra and other molecular methods was evaluated against a composite reference standard (CRS) comprising confirmed and presumed TB cases (Fig. 1).

### 2.3 Case definition

Confirmed TB lymphadenitis was defined by clinical or radiological evidence of lymphadenitis, accompanied by a positive microbiological test (either acid-fast bacilli culture or molecular assay) and absence of other etiology that would explain the diagnosis. Presumed TB lymphadenitis was defined by the presence of clinical or radiological signs of lymphadenitis with histopathological features indicative of tuberculosis (such as granulomatous or necrotizing granulomatous inflammation), a negative microbiological test, absence of other etiology that would explain the diagnosis, and a response to anti-tuberculous therapy.

### 2.4 Data collection

The BESTCare electronic system (ezCareTech, South Korea) was used to screen the electronic records of all patients who met the inclusion criteria. The following data were collected: demographic characteristics (age, gender, nationality), previous history of tuberculosis, and HIV status. Lymph node involvement was assessed by number, size, and site, with evaluation for extra-nodal disease. Clinical symptoms and results of lymph node investigations, including TB PCR, culture, and histopathology with acid-fast bacilli (AFB) staining, were also recorded.

### 2.5 Specimen collection and handling

Lymph node specimens were obtained through core needle biopsy, excisional biopsy, or fine-needle aspiration depending on clinical judgment and accessibility of the lymph node. In patients with multiple lymph nodes involved, the largest and most easily accessible lymph node was selected for diagnostic sampling by the interventional radiology or surgical team, and an average two to four passes were obtained during the biopsy procedure. The collected specimens were distributed for histopathological examination, acid-fast bacilli (AFB) staining, mycobacterial culture, and molecular testing depending on sample availability and clinical request. Not all specimens underwent all diagnostic tests due to variations in sample quantity and clinical practice during the study period. When multiple lymph nodes were involved, diagnostic testing was performed on the most accessible lymph node.

### 2.6 Specimen processing and molecular testing

Lymph node tissue specimens were processed in the microbiology laboratory according to institutional protocols. Briefly, tissue samples were aseptically minced and homogenized in sterile saline using a tissue grinder. When purulent material was present or contamination was suspected, the homogenized suspension underwent standard N-acetyl-L-cysteine–sodium hydroxide (NALC–NaOH) digestion and decontamination. The suspension was vortexed, incubated at room temperature for 15 min, diluted with phosphate buffer, and centrifuged at 3,000 × g for 20 min to obtain a concentrated sediment. The resulting sediment was used for acid-fast bacilli (AFB) smear microscopy and mycobacterial culture according to routine laboratory procedures.

Molecular testing for detection of *Mycobacterium tuberculosis* complex was performed using one of three nucleic acid amplification assays depending on the testing period and laboratory availability: Xpert MTB/RIF Ultra (Cepheid, USA), Xpert MTB/RIF (Cepheid, USA), or BD ProbeTec ET (Becton Dickinson, USA). For Xpert assays, processed specimen material was mixed with the sample reagent according to the manufacturer’s instructions and loaded into single-use cartridges for automated real-time PCR analysis in the GeneXpert system. These assays target specific genomic sequences of *Mycobacterium tuberculosis*, with Xpert MTB/RIF Ultra additionally targeting multicopy insertion sequences (IS6110 and IS1081) to enhance analytical sensitivity.

### 2.7 Statistical analysis

Statistical analysis was conducted using IBM SPSS version 27. Categorical data were analyzed using frequencies and percentages. Comparison between categorical data was assessed using the Chi-square test, with a p-value of less than 0.05 considered statistically significant. In addition, we calculated the sensitivity of GeneXpert MTB/RIF Ultra by defining false negatives in comparison against the composite reference standard and positive mycobacterial cultures.

### 2.8 Ettthical approval

This study was approved by the Institutional Review Board of the King Abdullah International Medical Research Center (KAIMRC); approval number 00000101225. Access to data was restricted to the researchers. The confidentiality of all patients was maintained, and no names or medical record numbers were used. All hard and soft copies of data were stored safely with limited access within the Ministry of National Guard-Health Affairs premises.

## 3 Results

A total of 85 cases of tuberculous lymphadenitis were identified and included in the composite reference standard, of which 35 were microbiologically confirmed and 50 were presumed cases. The most common age group was between 41 and 65 years of age (36.5%). The male gender was predominant (55.29%), and the majority of patients' nationalities were Saudi (91.76%). Over half of patients were diagnosed in inpatient settings (56.47%), and around 6% of them had a previous history of TB, and 2% were HIV positive.

The main presenting symptom was swelling (42.35%), followed by night sweats (22.35%) and weight loss (21.18%). Cervical lymph nodes were the most commonly involved (64.71%), followed by inguinal lymph nodes (16.47%). Most patients had more than four lymph nodes involved (45.88%), with a small size ranging between 1 and 2 cm (55.29%). Extra nodal involvement was identified in 28.24% of patients, with the lungs being the most commonly affected site (16.47%). Refer to Table 1 for further details.

**Table 1:** Baseline characteristics of patients with tuberculous lymphadenitis included in the composite reference standard.

**Demographics**	**N = 85 (%)**
**Age**	
18-25 yrs	6 (7.06)
26-40 yrs	24 (28.24)
**41-65 yrs**	**31 (36.47)**
> 65 yrs	24 (28.24)
**Saudi**	**78 (91.76)**
**Male**	47 (55.29)
**HIV Status**	
Positive	2 (2.35)
Negative	66 (77.65)
Not available	17 (20)
**Number of Lymph node involvement**	
1-2	31 (36.47)
3-4	15 (17.65)
**>4**	**39 (45.88)**
**Site of lymph node**	
Axillary	8 (9.41)
**Cervical**	**55 (64.71)**
Inguinal	14 (16.47)
Supraclavicular	2 (2.35)
Mediastinal, retroperitoneal, mesenteric	6 (7.06)
**Extranodal Involvement**	**24 (28.24)**
**Pulmonary Involvement**	**14 (16.47)**
Cardiac Involvement	1 (1.18)
Bone and Joint	3 (3.53)
Urogenital	1 (1.18)
CNS	3 (3.53)
Peritoneal	2 (2.35)
Pancreas	1 (1.18)
Bone Marrow	2 (2.35)
**TBL diagnosis**	
Confirmed TBL	35 (41.18)
**Presumed TBL**	**50 (58.82)**

HIV: human immunodeficiency virus, CNS: Central Nervous System, TBL: Tuberculosis lymphadenitis.

Most samples were obtained through core needle biopsy, which was performed in 76 patients (89.41%), followed by excisional biopsy in 8 patients (9.41%), and one patient underwent fine needle aspiration (FNA) (1.18%). The most common pathological finding was necrotizing/caseating granuloma (78.82%), followed by non-necrotizing/non-caseating granuloma (15.29%). Five samples were negative for granuloma/non-diagnostic but were positive for TB PCR Xpert-Ultra.

Acid-fast staining and cultures were performed on 81 and 39 samples, respectively. Only 12 samples were positive for AFB (14.81%), and 17 out of 39 cultures performed were positive (43.59%). Molecular testing was conducted using various PCR methods throughout the study period. Xpert-Ultra was performed on 63 samples (74.12%), while GeneXpert was done on 19 samples (22.35%), and ProbTec was performed on three samples (3.53%).

The overall PCR sensitivity, when compared to the composite reference standard, was 38.82% (33/85). Involvement of more than four lymph nodes and culture positivity were significantly associated with positive PCR results and positive Xpert-Ultra; no other factors were identified as being associated with PCR positivity or Xpert-Ultra positivity. (Table 2, Table 3).

**Table 2:** Factors associated with PCR positivity.

**Factors**	**Negative PCR N = 52 (%)**	**Positive PCR N = 33 (%)**	**P value**
**Age**			
18-25	3 (5.77)	3 (9.09)	0.574289
26-40	16 (30.77)	8 (24.24)	0.581023
41-65	18 (34.62)	13 (39.39)	0.722194
>65	15 (28.85)	9 (27.27)	0.894158
**Saudi**	49 (94.23)	29 (87.88)	0.765753
**Male**	29 (55.77)	18 (54.55)	0.941054
**Previous history of TB**	4 (7.69)	1 (3.03)	0.387771
**HIV Positive**	1 (1.92)	1 (3.03)	0.745692
**Number of lymph node involvement**			
1-2	27 (51.92)	4 (12.12)	0.003064
3-4	14 (26.92)	1 (3.03)	0.010603
**>4**	**11 (21.15)**	**28 (84.85)**	**0.000024**
**Site of lymph node**			
Mediastinal, retroperitoneal, mesenteric	3 (5.77)	3 (9.09)	0.574289
Axillary	6 (11.54)	2 (6.06)	0.422393
Cervical	31 (59.62)	24 (72.73)	0.463930
Inguinal	11 (21.15)	3 (9.09)	0.181709
Supraclavicular	1 (1.92)	1 (3.03)	0.745692
**Size of lymph node**			
<1cm	2 (3.85)	0	0.482699
1-2 cm	31 (59.62)	16 (48.48)	0.501232
2-3 cm	15 (28.85)	9 (27.27)	0.894158
3-4 cm	3 (5.77)	6 (18.18)	0.086537
>4 cm	0	2 (6.06)	0.164979
NA	1 (1.92)	0	0.619636
**Extranodal involvement**	12 (23.08)	12 (36.36)	0.261228

**Factors**	**Negative PCR N=52 (%)**	**Positive PCR N=33 (%)**	**P value**
**Specimen type**			
Core Needle	47 (90.38)	29 (87.88)	0.905220
Excisional	5 (9.62)	3 (9.09)	0.938772
FNA	0	1 (3.03)	0.326183
**Pathology**			
Not-diagnostic	0	3 (9.09)	0.029688
Caseating/Necrotizing Granuloma	42 (80.77)	25 (75.76)	0.799781
Negative for granuloma	0	2 (6.06)	0.075856
Non Caseating/Non Necrotizing Granuloma	10 (19.23)	3 (9.09)	0.244027
**PCR type**			
GenXpert Ultra	37 (71.15)	26 (78.79)	0.690320
GenXpert	13 (25.00)	6 (18.18)	0.517009
ProbeTec	2 (3.85)	1 (3.03)	0.845297
**Culture result**			
NA	31 (59.62)	15 (45.45)	0.387091
**Negative**	**19 (36.54)**	**3 (9.09)**	**0.015346**
**Positive**	**2 (3.85)**	**15 (45.45)**	**0.000029**
**AFB stain**			
NA	0.00	4 (12.12)	0.049569
Negative	44 (84.62)	25 (75.76)	0.658682
Positive	8 (15.38)	4 (12.12)	0.696354

PCR: Polymerase Chain Reaction, TB: Tuberculosis, HIV: human immunodeficiency virus, NA: Not available.

PCR: Polymerase Chain Reaction, FNA: Fine Needle Aspiration, NA: Not applicable, AFB: Acid-Fast Bacillus.

**Table 3:** Factors associated with Xpert MTB/RIF Ultra positivity.

**Factors**	**Negative Xpert MTB/RIF Ultra N = 37 (%)**	**Positive Xpert MTB/RIF Ultra N = 26 (%)**	**P value**
**Age**			
>65	11 (29.73)	8 (30.77)	0.941037
18-25	3 (8.11)	2 (7.69)	0.954008
26-40	11 (29.73)	7 (26.92)	0.837430
41-65	12 (32.43)	9 (34.62)	0.882542
**Saudi**	34 (91.89)	24 (92.31)	0.986489
**Male**	19 (51.35)	15 (57.69)	0.735899
**Previous history of TB**	4 (10.81)	1 (3.85)	0.334016
**HIV Positive**	1 (2.70)	1(3.85)	0.801985
**Number of lymph node involvement**			
**1-2**	**20 (54.05)**	**3 (11.54)**	**0.005967**
**3-4**	**10 (27.03)**	**1 (3.85)**	**0.030173**
**>4**	**7 (18.92)**	**22 (84.62)**	**0.000154**
**Site of lymph node**			
Mediastinal, retroperitoneal, mesenteric	3 (8.11)	1 (3.85)	0.508647
Axillary	5 (13.51)	1 (3.85)	0.220911
Cervical	23 (62.16)	21 (80.77)	0.384279
Inguinal	6 (16.22)	2 (7.69)	0.349933
Supraclavicular	0	1 (3.85)	0.232898
**Size of lymph node**			
<1cm	2 (5.41)	0.00	0.446309
1-2 cm	21 (56.76)	13 (50.00)	0.719291
2-3 cm	12 (32.43)	7 (26.92)	0.695041
3-4 cm	2 (5.41)	4 (15.38)	0.206375
>4 cm	0.00	2 (7.69)	0.196051
**Extranodal involvement**	9 (24.32)	8 (30.77)	0.627805
**Specimen type**			
Core Needle	33 (89.19)	22 (84.62)	0.848301
Excisional	4 (10.81)	3 (11.54)	0.932021
FNA	0.00	1 (3.85)	0.360608
**Pathology**			
Non-diagnostic	0.00	3 (11.54)	0.113318
Caseating/Necrotizing Granuloma	28 (75.68)	19 (73.08)	0.906407
Negative for granuloma	0.00	2 (7.69)	0.196051
Non Caseating/Necrotizing Granuloma	9 (24.32)	2 (7.69)	0.119856
**Culture result**			
NA	20 (54.05)	11 (42.31)	0.512886
**Negative**	**15 (40.54)**	**3 (11.54)**	**0.033988**
**Positive**	**2 (5.41)**	**12 (46.15)**	**0.000731**
**AFB stain**			
NA	0.00	4 (15.38)	0.067487
Negative	30 (81.08)	19 (73.08)	0.722849
Positive	7 (18.92)	3 (11.54)	0.469135

TB: Tuberculosis, HIV: human immunodeficiency virus, FNA: Fine Needle Aspiration, NA: Not applicable, AFB: Acid-Fast Bacillus.

Xpert-Ultra sensitivity was 41.27% (26/63), while GeneXpert was 31.58% (6/19), and ProbTec was 33.33% (1/3). When compared to culture positivity, Xpert-Ultra failed to detect two cases of culture-positive lymphadenitis but was positive in 3 culture-negative cases. No discrepancy was observed between the culture and GeneXpert or ProbTec (Table 4).

**Table 4:** PCR and Culture Discrepancy.

**PCR**	**Culture Positive N = 17 (%)**	**Culture Negative N = 22 (%)**
PCR Sensitivity	15/17 (88.24)	3/22 (13.64)
GeneXpert − Ultra	12/14 (85.71)	3/18 (16.6)
GeneXpert	3/3 (100)	0/3 (0)
ProbTec	−	0/1 (0)

PCR: Polymerase Chain Reaction.

Acid-fast bacilli were identified on histopathology in 12 cases. Among these, molecular testing was positive in 4 cases (3 by Xpert-Ultra and 1 by GeneXpert), while 8 cases had negative PCR results (7 Xpert-Ultra and 1 GeneXpert). Among the 69 cases with negative AFB staining, PCR testing was positive in 25 cases (19 Xpert-Ultra, 5 GeneXpert, and 1 ProbeTec), while 44 cases had negative PCR results (30 Xpert-Ultra, 12 GeneXpert, and 2 ProbeTec).

## 4 Discussion

The characteristics of our study population align with those reported in previous national studies. It consisted mainly of Saudi individuals between the ages of 41 and 65 years (36.5%), consistent with a retrospective study conducted over 20 years, showing that TB incidence increases with advancing age, with the highest rates observed in those older than 45 years. [17] Similarly, a local study of EPTB reported the highest incidence among Saudis between the ages of 25 and 64, further placing our results within commonly reported age ranges [18].

Gender distribution in our study mirrors both national and global patterns. Males accounted for 55.29% of the study population. This agrees with local data indicating higher TB prevalence in men than in women. [17], [18] Global estimates reported by the WHO are also consistent with our data, reporting that approximately 55% of total TB cases occur in adult males [1].

HIV co-infection was uncommon in our study population, accounting for only 2%. This low prevalence is consistent with the local estimates, which showed a prevalence of 3.2–3.5% for HIV co-infection, and with data from other low HIV prevalence countries. [2], [19], [20] However, this contrasts with the global data, as demonstrated by a multi-region *meta*-analysis reporting a pooled TB/HIV co-infection of 23.5%, with higher rates in Africa (31.25%) and Latin America (25.06%) and lower rates in the USA (14.84%), and Asia (17.21%; studies from Cambodia, India, Singapore, Thailand, Vietnam, and Iran) [21].

Enlarged lymphadenopathy, particularly in the cervical region, was the most common presenting symptom in our study (64.71%). Studies from Tunisia and Denmark demonstrated similar patterns, where 55.2% and 67.5% of patients presented with enlarged lymphadenopathy, and cervical predominance of 83.4% and 69.9%, respectively. [5], [20] Another local study also concurs with this finding, as most patients had enlarged lymph nodes with a rate of 72.8% cervical involvement. [8] Systemic symptoms were comparatively less common in our study (night sweats 22.35%, weight loss 21.18%), in contrast to Malaysian data, where systemic symptoms exceeded lymph node swelling as the main presentation among TB lymphadenitis patients (cough 73.39%, fever 53.21%, and night sweats 58%). [22] However, across these reports, the cervical region remains the predominant site of involvement [5], [20], [21], [22].

Tissue biopsy was the most commonly used approach at our institution during the study period. The predominant technique was core needle biopsy (89.41%), followed by excisional biopsy (9.41%), with only a single FNA sample collected (1.18%). This contrasts with the studies from Tunisia and Denmark, where excisional biopsy was the predominant method with rates of 72% and 36.1%, respectively [5], [20].

In this study, PCR testing demonstrated an overall moderate diagnostic yield compared to clinical diagnosis for TB lymphadenitis. We found an overall PCR sensitivity of 38.82% (33/85). When stratified by platform, Xpert-Ultra showed a sensitivity of 41.27% (26/63), while GeneXpert and ProbTec showed sensitivities of 31.58% (6/19) and 33.33% (1/3), respectively. This indicates that Xpert-Ultra achieved the highest yield among PCR platforms in our study. Nevertheless, across studies that used a composite reference standard (CRS), reported Xpert-Ultra sensitivities were often higher than ours. A recent prospective study reported Xpert-Ultra sensitivity of 91.66% in lymph node TB. [15] Another study evaluating Xpert-Ultra performance in TB adenitis showed sensitivities of 70% for FNA and 67% for tissue, highlighting slight differences between the two sampling methods. [23] However, both of those studies were conducted in countries with high endemicity of tuberculosis, which might explain the discrepancy with our study. Culture demonstrated a sensitivity of 43.6% (17/39) among the tested samples in our study. This is consistent with the culture sensitivity rates typically observed for TB lymphadenitis in the literature. A prospective TB adenitis study reported culture sensitivities of 33% (4/12) for FNA and 39% (9/23) for tissue biopsies, which are broadly comparable to ours, noting differences in specimen type. [23] Similarly, another study reported a culture sensitivity of 30.7% in TB lymphadenitis. [24] In contrast, a local study reported a higher culture sensitivity of 67.3% in lymph node tissue samples. [8] AFB staining exhibited the poorest performance in our study, with a positivity rate of 14.8% (12/81), consistent with the low estimates typically reported for TB lymphadenitis. [15], [18], [24] Because these different PCR platforms were applied to different specimens at different times in our study, direct head-to-head comparisons cannot be definitely drawn to conclude the superior sensitivity of Xpert-Ultra compared to other platforms.

Our study showed that Xpert-Ultra sensitivity compared with culture was 85.71%, among specimens with paired culture testing. Several studies have also evaluated the diagnostic performance of Xpert-Ultra in comparison to traditional culture methods. A study on the diagnostic accuracy of Xpert-Ultra reported sensitivities of 90.0% for lymph node tissue and 78.0% for FNA against culture, which approximates our results. [23] A large local series on EPTB reported an overall Xpert-Ultra sensitivity of 91% when compared to culture. [18] Other multicenter EPTB data show an overall sensitivity of Xpert Ultra of 80.66% and 87.50% for lymph nodes specifically (88.10% for biopsy and 86.36% for FNA) compared to culture. [16].

Overall, Xpert-Ultra did not outperform culture in our study, indicating non-superiority and reinforcing culture’s role as the gold standard, with Xpert-Ultra providing complementary and rapid detection. Several factors may explain this discrepancy. First, although Xpert-Ultra incorporates expanded reaction chambers and multicopy targets (IS6110 and IS1081), lowering its detection threshold to approximately 15.6 CFU/mL relative to the earlier assay Xpert MTB/RIF (131.0 CFU/ml), bacillary load can still fall below these limits especially in paucibacillary disease or when bacilli are heterogeneously distributed within the lymph node tissue. [12], [25] In line with this, Mekkaoui et al. observed that Xpert-Ultra false-negative results were associated with a low bacterial burden, as reflected by a prolonged time-to-culture positivity [26].

Second, our false-negative cases were obtained by core-needle biopsy. Although Xpert-Ultra showed a 94% specificity, a multicenter study by Maynard-Smith et al. found that its sensitivity in tissue specimens was only 50%, highlighting the possibility of false negatives in core or excisional biopsies. [27] The difficulties of applying molecular diagnostics to solid tissue specimens were further highlighted in a prospective head-to-head cohort study, which showed tissue-based Xpert-Ultra had a sensitivity of 73.6%, lower than the 81% reported for fine-needle aspirates. [13] Although previous studies reported higher sensitivity for lymph node tissue and that FNA when compared to positive cultures, the sensitivity of Xpert-Ultra from tissue specimens drops from 90% to 67% when compared to a composite reference standard. [23] Reported differences between tissue and FNA performance vary by setting; our reliance on core-needle biopsies likely contributed to the lower yields compared to studies with greater FNA use.

Finally, the epidemiological setting may have contributed to our findings. Saudi Arabia is classified by the WHO as a low-burden TB country, with an incidence of 8.4 (7.6–9.3) per 100,000 population. [1] In addition, extrapulmonary TB and non-tuberculous mycobacterial (NTM) infections are increasingly reported in the region. [28] Consequently, the incremental diagnostic yield of Xpert-Ultra may be limited, as the lower pre-test probability of TB, combined with a higher prevalence of atypical and NTM cases, reduces both its sensitivity and predictive value. [29].

This study has several limitations, including its retrospective single-center design and relatively small sample size, which limit its generalizability. The variability in diagnostic approaches across the study period, including the use of different molecular tests, and the lack of concomitant testing of the same samples with different tests, may limit the comparison between molecular tests. Since not all specimens were tested with each diagnostic platform in parallel, observed sensitivity differences may be driven by sampling and timing factors and should be interpreted accordingly. Lastly, cultures were performed in less than half of the patients, restricting direct head-to-head comparisons between Xpert-Ultra and culture. Future studies should use larger, prospective, multicenter cohorts and evaluate optimized sampling strategies, including parallel FNA and core-needle biopsy when feasible, to allow more precise comparison of diagnostic sensitivity between methods and possible exploration of other molecular techniques that would improve the yield of detection and lessen the empiric therapy.

## 5 Conclusion

This retrospective study evaluated the diagnostic accuracy of the Xpert-Ultra assay in identifying tuberculous lymphadenitis in a low TB-burden setting. Our findings reinforce the ongoing diagnostic challenge posed by the paucibacillary nature of TBL, as evidenced by the modest sensitivity of PCR-based methods and the traditional techniques such as smear microscopy and culture. Xpert-Ultra detected more cases of tuberculous lymphadenitis than the other molecular assays used in this study; however, direct comparison of diagnostic sensitivity between assays was not possible because the tests were not performed on the same specimens. Notably, the test failed to detect some culture-positive cases but identified a few cases missed by culture, highlighting its complementary role rather than a definitive replacement for traditional diagnostics. Despite the potential utility of Xpert-Ultra, its benefit remains moderate in a disease like tuberculous lymphadenitis. Further diagnostics are needed to obtain a confirmatory diagnosis rather than relying on a presumptive one.

## CRediT authorship contribution statement

**Manar Almutairi:** Writing – review & editing, Writing – original draft, Visualization, Validation, Supervision, Project administration, Methodology, Data curation, Conceptualization. **Reema Alalmaee:** Writing – review & editing, Writing – original draft, Validation, Project administration, Methodology, Data curation, Conceptualization. **Elaf Alamer:** Writing – review & editing, Writing – original draft, Validation, Project administration, Methodology, Data curation, Conceptualization. **Shaden Alharbi:** Writing – review & editing, Writing – original draft, Validation, Project administration, Methodology, Data curation, Conceptualization. **Gadah Aljarallah:** Writing – review & editing, Writing – original draft, Validation, Project administration, Methodology, Data curation, Conceptualization. **Raghad Almukhayzim:** Writing – review & editing, Writing – original draft, Validation, Project administration, Methodology, Data curation, Conceptualization. **Nawaf Albuhayjan:** Writing – review & editing, Writing – original draft, Validation, Supervision, Project administration, Methodology, Data curation, Conceptualization. **Reem Abanamy:** Writing – review & editing, Writing – original draft, Visualization, Validation, Supervision, Project administration, Methodology, Formal analysis, Data curation, Conceptualization.

## Funding

This research did not receive any specific grant from funding agencies in the public, commercial, or not-for-profit sectors.

## Declaration of competing interest

The authors declare that they have no known competing financial interests or personal relationships that could have appeared to influence the work reported in this paper.
